# Comparison of swarming, mating performance and longevity of males *Anopheles coluzzii* between individuals fed with different natural fruit juices in laboratory and semi-field conditions

**DOI:** 10.1186/s12936-020-03248-y

**Published:** 2020-05-06

**Authors:** Charles Nignan, Abdoulaye Niang, Hamidou Maïga, Simon Péguédwindé Sawadogo, Bèwadéyir Serge Poda, Olivier Gnankine, Kounbobr Roch Dabiré, Frédéric Tripet, Abdoulaye Diabaté

**Affiliations:** 1 Institut de Recherche en Sciences de la Santé, Bobo-Dioulasso, Burkina Faso; 2 Laboratoire d’Entomologie Fondamentale et Appliqué/UFR‑SVT/Université Joseph KI - ZERBO, Ouagadougou, Burkina Faso; 3 Centre for Applied Entomology and Parasitology, School of Life Sciences, Keele University, Keele, UK

**Keywords:** Malaria, Mosquito release, Sugar feeding, Trophic preference, Vector control

## Abstract

**Background:**

It is assumed that malaria vectors feed on locally available nectar sources to obtain energy. Sugar feeding is energetically critical for the *Anopheles* male swarming and mating activities. However, little is known about the impact of local nectar feeding on male physiological development and its consequences on male mosquito life traits in the malaria control context. This study aimed to evaluate the influence of local fruit juices on the life traits of males *Anopheles coluzzii*.

**Methods:**

Swarming characteristics (number of males in swarm, number of mating pairs, and swarm duration) in semi-field conditions; mating rate and longevity in a laboratory setting were compared between males *An. coluzzii* fed exclusively with mango, papaya or banana juices. The trophic preference was investigated in semi-field conditions.

**Results:**

The results of this study showed that in the laboratory, mosquitoes fed with papaya juices lived on average longer (10 days) than those fed with banana or mango juices (5 days) and had higher a mating rate (53%) than those fed with banana juice (40%). In the semi-field, the swarm size of mosquitoes fed with banana juice (85 males) was larger than that of mosquitoes fed with mango juice (60 males). The number of mating pairs formed from banana-fed male swarms (17 mating pairs) was higher than that formed from mango-fed male swarm (8 mating pairs). There was no difference in swarming duration between male treatments. Male mosquitoes had a preference for papaya and banana juices.

**Conclusions:**

The results indicate that the origin of plant-derived feeding is an important factor in the survival and reproduction of mosquitoes. This calls for further investigations of chemical contents of nectars and their impact on the physiological development of mosquitoes.

## Background

Malaria is a global health problem with sub-Saharan Africa being particularly badly hit by this disease. In 2018, 228 million estimated cases of malaria occurred worldwide with 213 million (93%) cases in Africa [1]. Well over 40% of the world’s population lives in these malaria endemic regions [1]. About 90% of the people affected are the poor and less privileged [2]. Children under 5 years of age and pregnant women are the most vulnerable groups affected by malaria [3].

Currently the most widely implemented vector control strategies are indoor residual spraying (IRS) and long-lasting insecticidal nets (LLINs), both of which have proven to be effective in reducing malaria transmission in some areas [4]. LLINs could achieve up 90% of reduction in the transmission rate if used by entire populations in these areas [4]. However, the emergence and rapid expansion of multiple insecticide resistance in major malaria vectors in sub-Saharan Africa, significantly undermine the effectiveness of IRS/LLINs [5, 6]. Despite significant progress in the development of candidate vaccines such as RTS,S/AS01 [7], no malaria vaccine is yet available. Complementary methods to be used alongside existing tools are urgently needed for more effective control of resistant vector populations.

The use of techniques like the Sterile Insect Technique (SIT) or Genetically Modified Mosquitoes (GMM) releases, as part of the Integrated Vector Management (IVM) tools are promising prospects. The SIT programme aims to reduce vector population density through releases of great number of laboratory-reared, sterile male mosquitoes that must successfully compete with wild males to mate with wild females. Females inseminated by sterile males lay unfertilised eggs that do not result in offspring [8–10]. The GMM focuses on mosquito population suppression or replacement by introducing genes of interest into the natural mosquito populations a progress of gene drive [11]. However, availability of these tools does not necessarily guarantee the success of vector control programmes. The control strategy can work only if the sterile or engineered males can successfully mate in the field. Thus, the ability to produce and release sexually competitive males is a critical aspect to the success of any mosquito release programme [12, 13]. Past failures in releasing sterile males [14] have led to repeated calls for further investigations into the behaviour and physiology of male mating [15, 16]. Careful selection of mating characteristics during colonization and rearing prior to release must be combined with intensive field trials to ensure phenotypic characters of released males are not antagonistic to longevity, dispersal or mating competitiveness [17].

*Anopheles gambiae* sensu lato (*s.l*.) mate in flight. Males aggregate in swarms at sunset waiting for females to copulate with them [18, 19]. Females visit these aggregations, find a male and the mating pairs leave the swarm in *copula* [19–21]. Swarming activity lasts about 22 min in the field [22] and consumes about 50% of male’s sugar and glycogen reserves [23, 24]. Thus, the demand on energy reserves for swarming flight is very high [25, 26], and a crucial factor in ensuring reproductive success and survival. A male mosquito can mate more than once during its life time [27], suggesting the ability to replenish its energy reserve after each swarming activity, by feeding on natural sugar sources.

Nutritional reserves accumulated during larval development and from sugar-feeding are critical determinants of adult survival and mating success [26, 28–30]. Consequently, the success of male mosquito release programmes (SIT or GMM) strongly depends on the quality, availability and accessibility of foods for adult males in the fields. However, there is a lack of data on the impact of various natural sugar meals on the physiological development and life history traits of male mosquitoes. In this study, the trophic preference of males *An. coluzzii* and compared their swarming activity, mating performance and longevity between males fed with different natural fruit juices has been assessed under laboratory and semi-field conditions.

## Methods

### Mosquitoes

*Anopheles coluzzii* was used for all the experiments. This species is kept and reared in the insectary of the Institut de Recherche en Sciences de la Santé (IRSS) in Bobo-Dioulasso, Burkina Faso. This strain was established from the eggs obtained from wild gravid females *An. coluzzii* collected indoors at VK5 in 2008. For all the experiments, eggs from this laboratory-rearing *An. coluzzii* strain were allowed to hatch in tap water the larvae fed with Tetramin^®^ Baby Fish Food (Tetrawerke, Melle, Germany) ad libitum. Pupae were removed daily, and separated by sex under a stereomicroscope (Leica S6E) by examining the genital segment (*i.e.* the terminal segment) for sexual dimorphism [31]. Then, the male and female pupae were separated and transferred into small plastic cups (Ø = 45 mm, h = 85 mm; ≈ 200 pupae per cup), placed in 30 × 30 × 30 cm mesh covered cages and kept in the insectary under standard conditions (27 ± 2 °C, 75 ± 5% RH, 12L:12D) until emergence. All the mosquitoes used in an experiment came from the same batch.

### Fruit juices preparation

All the experiments, were carried out on three sweet fruit juices made from local fruits namely; banana (*Musa ssp.,* “poyo” variety), papaya (*Carica papaya*, “solo” variety) and mango (*Mangifera indica,* “lipince” variety). All these three fruits were available in the local market in Bobo-Dioulasso and from trees commonly found around human dwellings in the villages and cities of western Burkina Faso. Thus, it is assumed that *An. coluzzii* mostly feeds on them in their natural habitat.

For each juice, 100 grams of fresh fruit pulp was ground in an electric grinder until the pulp was completely shredded. Between each fruit, the grinder was cleaned with distilled water to avoid contamination. The juices were conserved in the refrigerator at + 4 °C until they were used for tests.

### Experimental design in the laboratory (longevity and mating performance)

#### Longevity

Upon emergence, four batches of 50 virgin male mosquitoes were put in 20 × 20 × 20 cm mesh covered cages. Then, each cage was randomly provided with one type of juice, either banana, mango or papaya, a fourth cage was provided with a 5% glucose solution and was used as a control. Indeed, 5% glucose solution is the sugar solution used to feed adult mosquitoes in our insectaries. The cages were placed in standard insectary conditions (27 ± 2 °C, 75 ± 5% RH, 12L:12D), the sugar meal pads were replaced daily with new ones and dead mosquitoes were removed daily and counted. Longevity was considered as the lifespan from the day of adult emergence to the day of death. Six replicates were carried out.

#### Mating performance

As in the previous experiment, upon emergence, four cohorts of 50 virgin males were put in 20 × 20 × 20 cm mesh covered cages, each provided with one type of juice, either, banana, mango or papaya and, the fourth cage provided with 5% glucose solution. The cages were placed in standard insectary conditions. 3 days later, twenty-five 3-days old virgin females, previously kept in cages provided with 5% glucose solution, were removed from their cages and put into each of the four cages containing the males. Thus, allowing mating ratios of 2:1 (males:females). Males and females were kept together for 3 days and then, all the females were removed from the cages. Their spermatheca was dissected under stereomicroscope (Leica S6E) and the insemination status was assessed under a compound light microscope (Leica DM750) at 400 × magnification to observe the presence/absence of spermatozoa. Five replicates were carried out.

### Experimental design in semi-field (trophic preference, swarming and mating performance)

The semi-field experiments were carried out in the Mosquito Ecology Research Facility (MERF) at VK7 in Bama (Burkina Faso) described by Niang et al. [32]. Briefly, the MERF is composed of 11 experimental compartments measuring 10 × 6 ×4.5 m (L × W × H) each, with a floor made of concrete, walls made of polyester net and a roof made of a transparent polyene; the whole ensuring climatic conditions similar to the surrounding ambient conditions and an optimal diffusion of daylight into the compartments.

#### Trophic preference

**Fig. 1:**
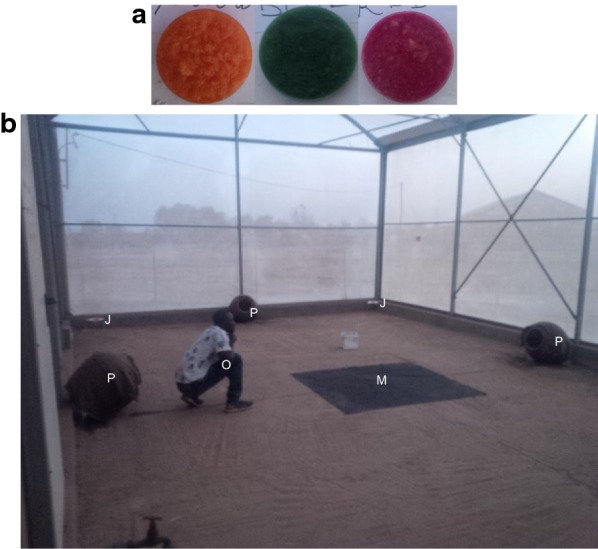
Experimental design for the trophic preference experiments in semi-field conditions. **a** Fruit juices coloured with food dyes of different colours. **b** Experimental design in an experimental compartment: visual marker (M), clay pot (P), tested juice (J), observer (O)

The three fruit juices (banana, mango and papaya) were distinctly coloured with food dyes (Fig. 1a) to help identify the origin of the sugar meal in the mosquito’s abdomen after feeding. In the evenings, the juices were put separately in petri dishes and placed in the corners of an experimental compartment (one juice pot per corner) (Fig. 1b). The juice pots were placed on supports 30 cm above the ground and at least 6 m from each other. Four clay pots used as mosquito resting sites, were placed close to the walls and halfway between two juice pots inside the experimental compartment, (Fig. 1b). The opening of the clay pots was directed horizontally towards the centre of the compartment (Fig. 1b). The clay pots contained a little wet sand and were covered with wet jute bags to increase the humidity and make them more attractive.

At sunset (≈ 18:00), a 1.5 × 1.5 m a black cloth, used as a visual marker to stimulate swarm formation (see [32, 33]), was placed at the centre of the experimental compartment (Fig. 1b) and three hundred 3-day old male mosquitoes were released into the experimental compartment. Swarming activity started about 15 min later and lasted about 22 min. The next morning, between 07:00 and 08:00, resting mosquitoes were collected from the clay pots using a mouth aspirator. Sugar feeding status and origin of all collected mosquitoes were determined through a visual examination of the abdomen. Seven replicates were carried out. The matching between fruit juice, colour and location was rotated between each replicate in order to avoid both colour and location bias.

#### Mating performance

In the evenings, 3-day old virgin males fed with the four different sugar meals (*i.e.* banana, mango, papaya juices and 5% glucose solution) since emergence and 3-days old virgin females fed only with 5% glucose solution were transported in 20 × 20 × 20 cm mesh covered cages to the MERF and kept inside the experimental compartments for acclimation. At sunset (≈ 18:00), 300 males of each treatment were released at the same time as 100 females into four experimental compartments (a treatment per compartment) following a mating ratio of 3:1 (males:females) per experimental compartment. Each compartment was provided with a 1.5 × 1.5 m black cloth on the floor that served as visual markers to stimulate swarm formation. Two highly trained observers per experimental compartment monitored the swarms and recorded data. For each swarm, the following parameters were recorded during swarming time: (i) the start time of swarming (*i.e.* when the first male started swarming), (ii) the estimated number of mosquitoes in the swarm 10 min after the start of swarming (*i.e.* the swarm size at the swarm peak time), (iii) the end time of swarming (*i.e.* when the swarm dispersed) and, (iv) the number of observed mating pairs during the all swarming time. Some observed mating pairs were caught using a collection net and the spermatheca of the females were dissected in the laboratory to assess their insemination status. Twelve replicates were carried out. The matching between male treatment and experimental compartment was rotated between each replicate in order to avoid compartment bias.

### Statistical analysis

All analyses were performed using R (version 3.5.2). The mosquito survivorship was analysed as a function of male treatment (fed with different fruit juices) using Cox’s proportional hazard regression models (“coxph” function in the “survival” package). The proportions of mosquito fed on the different fruit juices (trophic preference) were compared using a binomial Generalized Linear Model (GLM). The swarm size (*i.e.* the estimated number of males in swarm) as a function of male treatment was analysed using a GLM with a Poisson error. The swarm duration (*i.e.* the time elapsed between the start and the end of swarming activity) was analysed using a GLM with a Gaussian distribution, and male treatment, swarm size and their interactions were considered as fixed effects. The number of observed mating pairs was analysed using a GLM with a Poisson error, and male treatment, swarm size, swarm duration and their interactions were considered as fixed effects. The insemination rates (in laboratory and semi-field) were analysed as a function of male treatment using a binomial GLM.

For model selection, we used the stepwise removal of terms, followed by likelihood ratio tests (LRT). Term removals that significantly reduced explanatory power (*P *<* 0.05*) were retained in the minimal adequate model [34]. Post-hoc pairwise comparisons were done (“glht” function in “multcomp” package) with the Tukey method. Results are presented as mean ± standard error (se) and proportion ± 95% confidence interval (CI).

## Results

### Longevity and mating performance in laboratory

**Fig. 2:**
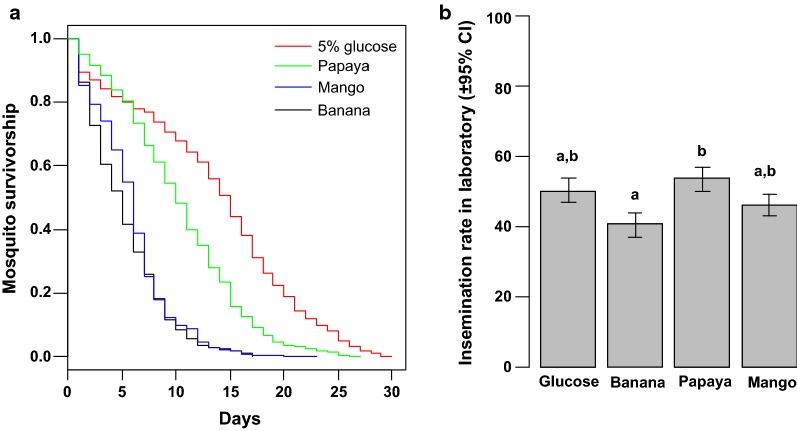
Comparison of mosquito survival and mating performance between males fed with the three fruit juices in laboratory conditions. **a** Male survivorship. **b** Female insemination rate. The bars with different letters on top were significantly different (*p *< 0.05)

Longevity —was a significant difference in mosquito survival between the different male treatments ($\chi_{3}^{2}$ = 20.54, *p *<0.001; Fig. 2a). The mosquitoes fed with 5% glucose solution or papaya juice lived longer than those fed with either banana or mango juices (on average 13.56 ± 0.3, 10.30 ± 0.2, 5.27 ± 0.1 and 5.85 ± 0.1 days, respectively).

Insemination rate—after keeping males and females together for 3 days in laboratory cages, an overall insemination rate of 47.40 ± 3.36% was found. There was a significant difference in insemination rate between the male treatments ($\chi_{3}^{2}$ = 8.5, *p* = 0.036; Fig. 2b). The insemination rate of females kept together with males fed with papaya juice was higher than that of females kept together with males fed with banana juice.

### Trophic preference and mating performance in the semi-field

**Fig. 3:**
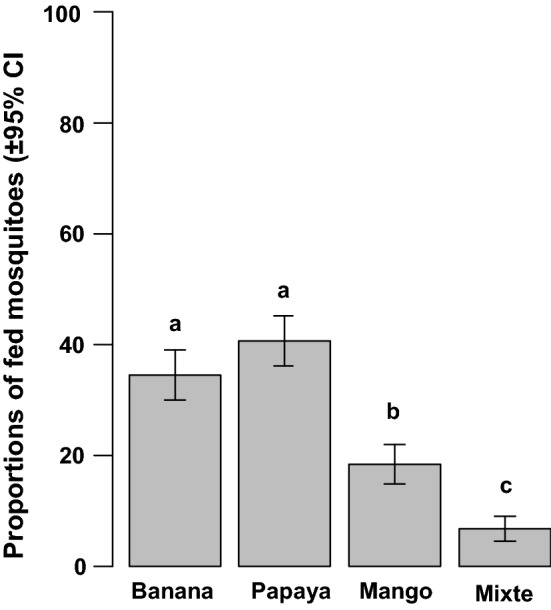
Mosquito trophic preference in semi-field conditions. Comparison of numbers of mosquitoes fed on tested fruit juices. The bars with different letters on top were significantly different (*p *< 0.05)

Trophic preference— a total of 513 (24.42%) mosquitoes were recaptured out of 2100 males released into the experimental compartment. Of these, 450 (87.72%) were fed on at least one of the three presented fruit juices and 63 (12.28%) were unfed. There was a significant difference in the proportions of mosquitoes that fed on the presented fruit juices ($\chi_{3}^{2}$ = 67.7, *p* < 0.001; Fig. 3). The proportions of mosquitoes that fed on banana juice and papaya juice were significantly higher than the proportion of mosquitoes fed on mango juice. There was a low rate (6.66 ± 2.30%) of mixed feeding (*i.e.,* mosquitoes fed on at least two fruit juices).

**Fig. 4:**
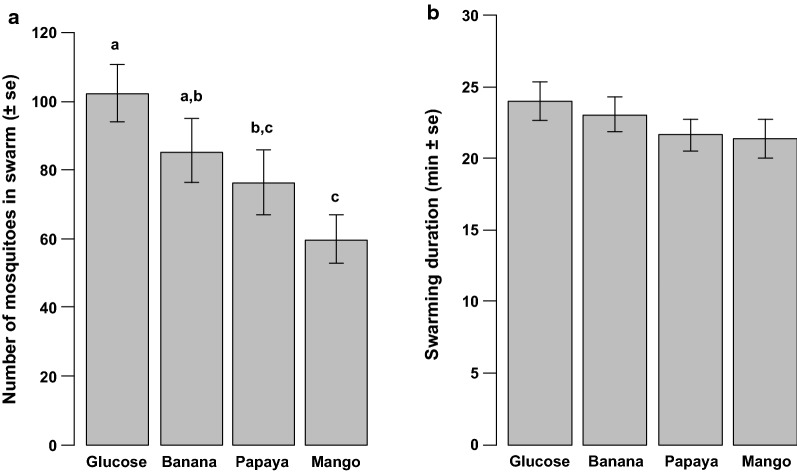
Comparison of swarm characteristics of males fed on the tested fruit juices. **a** Swarm size. **b** Swarming duration. The bars with different letters on top were significantly different (*p *< 0.05)

Swarm size (estimated number of males in the swarms)—an average number of 80.95 ± 4.13 male swarmed out of the 300 males released per test. There was a significant difference in the swarm size between the male treatments ($\chi_{3}^{2}$ = 19.1, *p *< 0.001; Fig. 4a). The swarm formed by mosquitoes fed with 5% glucose solution was significantly larger than the swarm of mosquitoes fed with papaya or mango juices; the swarm of mosquitoes fed with banana juice was significantly larger than the swarm of mosquitoes fed with mango juice.

Swarm duration—the swarming activity lasted an average of 22.54 ± 1.26 min. There was not significant difference in the swarming duration between male treatments ($\chi_{3}^{2}$ = 53.4, *p* = 0.43; Fig. 4b). No significant effect of swarm size on the swarming duration was found ($\chi_{1}^{2}$ = 0.2, *p* = 0.91) and there was no significant interaction between male treatment and swarm size on the swarm duration ($\chi_{3}^{2}$ = 81.4, *p* = 0.23).

**Fig. 5:**
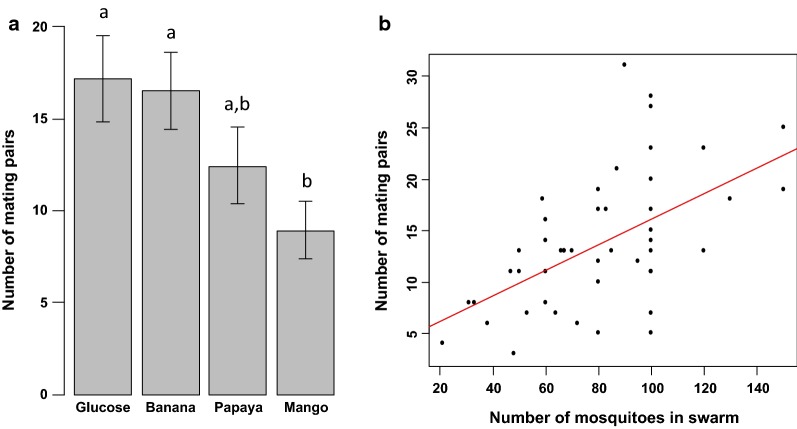
Comparison of swarm characteristics of males fed on the tested fruit juices. **a** Number of mating pairs. **b** Effect of swam size on number of mating pairs. The bars with different letters on top were significantly different (*p *< 0.05)

**Fig. 6:**
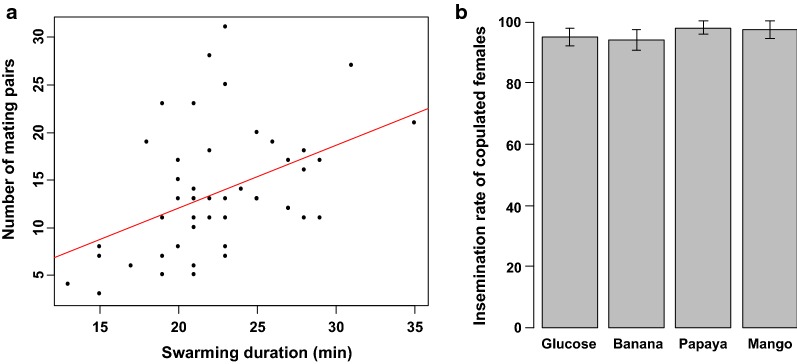
Comparison of swarm characteristics of males fed on the tested fruit juices. **a** Effect of swarming duration on number of mating pairs. **b** Female insemination rate

Number of mating pairs—an overall number of 660 mating pairs were collected out of 4 800 released females (13.75% of mating rate) during male swarming activities. There was a significant difference in the number of mating pairs between male treatments ($\chi_{3}^{2}$ = 13.3, *p *= 0.003; Fig. 5a). The numbers of mating pairs collected from 5% glucose-fed or banana-fed male swarms were significantly higher than those collected from mango-fed male swarm. There was a significant effect of swarm size (the number of mosquitoes in swarm) and swarm duration on the number of mating pairs collected ($\chi_{1}^{2}$ = 11.8, *p *< 0.001; $\chi_{1}^{2}$ = 20.7, *p *<0.001; Figs. 5b and 6a; respectively). More mating couples were collected from larger swarms or from swarms with a longer swarming time. No significant interaction between the effect of swarm size and swarm duration on the number of mating pairs was found ($\chi_{1}^{2}$ = 0.6, *p* = 0.55), nor was there an interaction between male treatment and swarm duration ($\chi_{3}^{2}$ = 4.0, *p* = 0.51) or swarm size and male treatment ($\chi_{3}^{2}$ = 5.12, *p* = 0.41). Of the 660 mating pairs collected from the swarms, 628 (95.15%) were inseminated. There was not significant difference in the insemination rates between male treatments ($\chi_{3}^{2}$ = 2.7, *p *=0.43; Fig. 6b).

## Discussion

Adult mosquitoes emerge with low energy reserves and build it up by sugar-feeding [35]. Thus, the nature and the origin of sugar are very important for mosquito life trait activities [26, 36]. Feeding on different natural sugar sources can influence the physiological development and consequently can affect the key life history traits. Here, we compared the longevity, swarming activity and mating performance of *An. coluzzii* between males fed with three natural fruit juices namely papaya, mango and banana. In this study, the results showed that the papaya juice produced males with greater longevity than banana and mango juices. Mosquitoes fed on banana or mango juices lived an average of 5–6 days in laboratory conditions. Moreover, a previous study in Burkina by Hien et al. found the same conclusions [37], and also they showed that mango nectar reduces *An. coluzzii* longevity of 5 to 6 days. The negative impact of some plant-derived sugar meal on mosquito survivorship could be explained by the presence of some toxic secondary metabolites (e.g. alkaloids, terpenes, glycosides) which play a role in the defence of plants against herbivore insects. For example, in honeybees, the ingestion of alkaloids contained in floral nectars could reduce bees longevity [38]. Banana and mango could have these toxic secondary metabolites in relatively higher proportions compared with papaya. This might also be the result of complex interactions between toxic secondary metabolites and the nutritional quality of the plant sugar meal.

The results of the current study showed that mosquitoes fed on mango juice were less likely to engage in swarming activity. This could potentially be a consequence of a physiological development unfavourable to competitive mating in a swarming system, or an insufficient energetic reserve for swarming activity. Although the criteria for mate selection within the “*lek*-like” system in which *Anopheles* mosquitoes mate are not yet well known [39], it is likely that morphological and physiological criteria are considered for the mate choice. It has been reported that in the swarms of *An. gambiae s.l.*, mated males were on average bigger than un-mated ones [23, 26], suggesting that the body size could be an important criterion for choosing a mate in *An. gambiae s.l.* species. This study does not allow us to conclude on the impact of the different fruit juice meals on mosquito size. However, given the importance of sugar meals in the biology of male mosquitoes [40, 41], one might suspect that mango juice may not contain nutrients needed for better development of males, and glucose, banana and papaya juices, which were associated with larger swarms, could ensure proper development of males. Glycogen and sugar reserves constitute the main energy fuel for mosquito flight [24]. Swarming activity has an important energy cost and consume about 50% of available energy [23, 26, 28, 41, 42]. Males that engage in swarming activities to find mates are assumed to have the required energy for swarming flight and mating. In this respect, these results may to suggest that the mango juice meal would not provide males with enough energy for several individuals to engage in a swarming activity compared with the papaya, banana and glucose juice meal. Unfortunately, this study could not to conclude on the energy reserve after feeding on the three studied fruit juices.

The swarming lasted an average of 22 min regardless of male treatment and swarm size. This supports the assumption that males engaging in swarming would have the required energy for swarming flight. A swarming activity consumes about 16% of the total sugars energy and a mosquito spends 5.9-fold as much sugar energy in swarming activities than when it is at rest [26]. Thus, the demand for energy is very high and only individuals able to satisfy this energy demand would form the swarm. The same swarming duration was reported for natural swarms of the same species [25], suggesting that when feeding exclusively on a fruit juice, male mosquitoes can produce a swarm as lasting as natural mosquitoes fed on a variety of plant-derived sugar sources.

Swarms formed by males fed with mango juice resulted in a low number of mating compared to other treatments. One could suggest that this is due to a physiological deficiency of these males when mating with females. It may be that the body size of the male individuals was not suitable for the females, studies have shown the importance of body size in mating success in *An. gambiae s.l.* [26, 30]. This may also be explained by a poor energetic reserve used by these males to mate with females. However, studies failed to show the importance of energetic reserve in mating success in *An. gambiae s.l.* by examining energy reserves in mated and un-mated males participating in swarming activities [30]. The results of this study showed that it could be an effect of swarm size (number of mosquitoes in the swarm). Many mating pairs were collected from the swarms formed by the males fed with 5% glucose solution, banana and papaya juices, which were also the swarms with many individuals. A strong correlation between swarm size and the number of mating pairs was observed in *An. gambiae s.l*. species in the field [39, 43]. This is consistent with the hotspot model of *lek* formation. Males aggregate in swarm at specific spots to increase their chance of encountering females, and females are likely to join large swarms to increase their chance of encountering “good quality” males.

Almost all females collected in mating pairs were successfully inseminated (95%) regardless male treatment. Similar insemination rates were reported in females collected in mating pairs from natural *An. gambiae s.l.* swarms [43, 44]. Feeding on any of the three studied fruit juices, the males in swarm successfully mated with females, suggesting that swarming males had required energy for swarming and mating competition. However, under laboratory conditions, differential mating success was observed; males fed with banana juice had relatively low success at mating compared to other treatments. Considering that the mating rate in natural and semi-field conditions is positively correlated with the number of mosquitoes that are able to form a swarm, one can assume that some of those mosquitoes physiologically unable to mate in a swarming system are able to mate when they are confined with females in laboratory cages. This could be explained by the fact that the males in the laboratory cage mate without the need to form swarm and, therefore, have a low energy requirement. It could also be due to the fact that the choice of the mate based on some criteria is limited in confined conditions. These could explain the fact that in our study, males fed with mango juice had a mating rate similar to that of males fed with papaya juice and 5% glucose solution in the laboratory conditions, but had formed a swarm of only a few individuals leading to a low number of mating pairs compared to other treatments in the semi-field conditions. This suggests that the swarm could serve as a first filter in sexual competition in the species mating in a *lek* system.

Males *An. coluzzii* fed preferentially on papaya and banana juices after swarming activities in our semi-field experimental conditions. In the field, sugar feeding by males occurred in the resting place before swarming, after swarming and at other times during the night [35]. Swarming is a crucial step in mosquito mating and consume a large proportion of male sugar and glycogen stores [23, 26, 28, 45]. Consequently, a sugar meal that would be both energetic and conducive to better physiological development is necessary after a swarming activity, not only to replenish energy stores but also to restore the physiological health of the mosquito’s body for the next swarming activity. Papaya juice meal provided better longevity and mating performance, and the banana juice meal also provided a good mating performance to the males compared to the mango juice in our experimental conditions. The preference for these two fruit juices suggests that mosquitoes could detect and recognize a “good” sugar meal. Mosquitoes have a highly developed olfactory sense, and the odours of plant-derived products could allow them to know the nutritional composition and influence on their behaviour. The results support the findings of Malmgren et al. [46] that have showed that mosquitoes were most attracted to the fruit of papaya. The mixed feedings observed in our semi-field experiment suggests that mosquitoes can take their sugar meal on many plant-derived sugar-sources in the field, and it would be important to take into account the influence of mixed feeding on mosquito life traits. Further studies of these fruit juices are needed to investigate the chemical composition, nutritional content and their proportion in order to better understand their influence on the life traits and physiological development of the mosquitoes feeding on them.

## Conclusion

This study showed that the origin of plant-derived sugar meal can influence the life traits of mosquitoes. The three fruit juices tested with males *An. coluzzii* showed a differential influence on mosquito life traits with individuals fed on mango or banana juices having a short life span and those fed on mango juice showing a poor mating performance. This suggests the nectar sources present in an area could be an important factor in the survival and reproduction of the mosquitoes in addition to some intrinsic and extrinsic factors. In this regard and according to the results of our study, in the context of male release for malaria control, the presence of banana and/or papaya plants in the release area would be beneficial to the programme. However, further investigations of the energy reserves of mosquitoes fed on the different fruit juices, the chemical composition and nutritional content of the fruit juices, as well as their impact on the physiological development of the mosquitoes, are needed for a better understanding of the sugar feeding ecology of the vectors in a context of effective malaria control.

## Data Availability

The raw datasets are available from the corresponding author on reasonable request.

## Abbreviations

SIT: Sterile insect technique
WHO: World Health Organization
RH: Relative humidity
LD: Light dark
IRSS: Institut de Recherche en Sciences de la Santé
VK: Vallée du Kou
MERF: Mosquito ecology research facility
